# Marked Antigiardial Activity of *Yucca baccata* Extracts: A Potential Natural Alternative for Treating Protozoan Infections

**DOI:** 10.1155/2014/823492

**Published:** 2014-08-31

**Authors:** Luis Quihui-Cota, Rocio León-Trujillo, Humberto Astiazarán-García, Julián Esparza-Romero, María del Refugio Robles, Ramón E. Robles-Zepeda, Rafael Canett, Jesús Sánchez-Escalante

**Affiliations:** ^1^Departamento de Nutrición Pública y Salud, Coordinación de Nutrición, Centro de Investigación en Alimentación y Desarrollo, A.C. Carretera a La Victoria Km 0.6, 83304 Hermosillo, SON, Mexico; ^2^Departamento de Nutrición y Metabolismo, Coordinación de Nutrición, Centro de Investigación en Alimentación y Desarrollo, A.C. Carretera a La Victoria Km 0.6, 83304 Hermosillo, SON, Mexico; ^3^Departamento de Ciencias de los Alimentos, Centro de Investigación en Alimentación y Desarrollo, A.C. Carretera a La Victoria Km 0.6, 83304 Hermosillo, SON, Mexico; ^4^Departamento de Ciencias Químico Biológicas, Universidad de Sonora, Bulevar Luis Encinas y Rosales s/n, 83000 Hermosillo, SON, Mexico; ^5^Departamento de Investigación y Posgrado en Alimentos, Universidad de Sonora, Bulevar Luis Encinas y Rosales s/n, 83000 Hermosillo, SON, Mexico; ^6^Herbario USON, Departamento de Investigaciones Científicas y Tecnológica, Universidad de Sonora, Niños Héroes entre Rosales y José María Pino Suárez, Edificio 1A (Museo), Colonia Centro, 83000 Hermosillo, SON, Mexico

## Abstract

Human Giardiosis is a public health problem in Mexico, where the national prevalence was estimated to be up to 68%. Misuse of antiprotozoal drugs may result in low effectiveness and undesirable side effects. Research on natural products is a good strategy for discovering more effective antiparasitic compounds. This study evaluated the antigiardial activity of extracts of* Yucca baccata*, which is native to northwestern Mexico. Forty-two gerbils (females) were weighed and orally inoculated with 5 × 10^6^ *Giardia* trophozoites. Two gerbils were selected at random to confirm infection. Forty living gerbils were randomly allocated into 5 treatment groups (8 per group). Gerbils were randomly assigned to be treated with 24.4 mg/mL, 12.2 mg/mL, and 6.1 mg/mL of extracts, metronidazole (2 mg/mL) or PBS, which were intragastrically administered once per day for 3 days. Nine gerbils died during the study course. On day 10 postinfection, gerbils were euthanized and trophozoites were quantified.* Yucca* extracts reduced, albeit not significantly, the trophozoite counts in the duodenum segment. Only the high-extract concentration significantly reduced the trophozoite counts in the proximal segment and it was similar to that of metronidazole. Extracts of* Y. baccata* may represent an effective and natural therapeutic alternative for human giardiosis.

## 1. Introduction

One of the most important intestinal protozoans responsible for human infections worldwide is* Giardia duodenalis*. In 2000, it was estimated that 200 million people in Asia, Africa, and Latin America showed symptomatic giardiosis and 500,000 new cases were diagnosed annually [1]. Later estimations showed that it could be responsible for one billion cases annually and is accompanied by an overall worldwide prevalence of 30% [2]. Acute symptoms include diarrhea, gas, abdominal cramps, nausea or vomiting, and dehydration [3, 4]. In some areas of Mexico, the prevalence of giardiosis can reach up to 68% [5] and now* G. duodenalis* is the most important protozoan parasite causing human intestinal infection in Northwestern Mexico [6]. Currently, this infection is treated with conventional and effective drugs (quinacrine, nitroimidazoles, and nitrofurans) [7]; however, people reject them because of their undesirable side effects [8]. Otherwise, a meta-analysis review [9] concluded that safety, effectiveness, and low cost make of albendazole an alternative and/or replacement for the metronidazole in the treatment of giardiosis in humans. However, the development of resistant strains due to overuse of any drug and the possibility of finding a drug to be used in shorter treatment times than those of the conventional drugs are some reasons for considering alternative therapies. One of them being investigated for the treatment of giardiosis consists of new antigiardialcompounds with different structures and mechanisms of action compared to conventional drugs. Since ancient times, people have used plants to treat common infectious diseases, and some of them have been integrated as a cure for diverse pathologies [10].* Yucca* was one of the plants used as an important natural resource by Indians of the American Southwest, Mexico, and Latin America for thousands of years against arthritis, fever, headaches, ulcers, and appendicitis [11]. Extracts of* Yucca* contain a variety of compounds such as saponins, alkaloids, tannin, terpenoids, and reducing sugars [12]. Saponins of* Yucca schidigera,* a plant of the family Agavaceae and native to the southwestern United States and Baja California in Mexico [13], have been investigated as defaunation agents. In addition, extracts of* Y. schidigera *have been shown to reduce the rumen protozoal population in vitro [14] and in vivo [15]. Based on that information, the present study was conducted to investigate whether extracts of another species,* Y. baccata,* which is native to the desert on the Mexican side (Northwestern Mexico), possess antigiardial activity and to consider it as a potential agent for controlling giardiosis could be potential agents for controlling giardiosis.

## 2. Materials and Methods

### 2.1. Plant Material

Samples of* Yucca baccata* were collected at the Rancho El Aribabi located in northeastern Sonora, Mexico. The samples were placed in bags and transported to the herbarium of the University of Sonora (Herbarium USON) for their taxonomic authentication. The material used in this study was backed up with a specimen of reference (USON 18607, J. Sánchez 2011-095) that is currently deposited in the USON collection.

### 2.2. *Yucca baccata* Extract Preparation

Once the* Yucca* stem was dried under sunny conditions (25°C–35°C), it was pulverized using a Wiley Mill (Thomas-Wiley Mill, Model 4, Laboratory Mill, USA) to obtain a coarse powder (2 mm particle size). The preparation of the extracts was performed based on the method described by Newbold et al. [16] for* Sesbania sesban*. An aqueous suspension of the pulverized material of the stem of* Y. baccata* (33 g/L) was left overnight at room temperature (25°C–30°C). The aqueous material was filtered and shaken in equal volumes of* n*-butanol for 30 minutes at room temperature. Extractions were repeated with* n*-butanol. The resulting organic layers from each extraction process were pooled and dried using a rotavapor (ROTAVAPOR BUCHI 461 Water Bath, Pace Analytical Services, Minneapolis, MN, USA) at 40°C, and the residue was weighed and resuspended in PBS at a 1 : 15 ratio. The solution was clarified by filtration through preweighed Whatman no. 1 filter paper. Solid residues were scraped from the filter paper and washed three times with* n*-butanol, and the liquid extractant was rotoevaporated. The residue was weighed and stored at 4°C in 15 mL centrifuge tubes prior to use in the preparation of extracts with 3 different concentrations before their administration to the treatment groups.

### 2.3. Animals

Forty-two female Mongolian gerbils of the strain* Meriones unguiculatus*, aged 6–12 weeks old and weighing 41 g–64 g, from the Animal Bioterium Center of the National Autonomous University of Mexico (acronym in Spanish UNAM), were used in this study. We decided to work with this number of gerbils on the basis of our previous findings with* Y. schidigera* [17]. The gerbil is susceptible to* G. duodenalis* infection [18].

### 2.4. Ethical Considerations

This study was approved by the ethical committee of the Centro de Investigación en Alimentación y Desarrollo A.C. (Centre of Research in Food and Development) to be carried out in strict accordance with the recommendations in the Guide for the Care and Use of Laboratory Animals of the Mexican Official Regulations [19] and the Committee on Care and Use of Laboratory Animals of the Institute of Laboratory Animals Resources [20]. All surgery was performed under chloroform anesthesia, and efforts to minimize animal suffering were made during the study course.

### 2.5. Bioterium Area Conditions

The bioterium was sanitized with sodium 10% hypochlorite. The temperature was maintained between 20°C and 26°C (mean 22°C ± 1°C) and humidity between 40% and 70%; there were 10 to 14 air-room changes per h and 12-12 h-light/dark cycles. The gerbils were housed in individual sanitized stainless-steel cages (60 × 40 × 30) and bedded on sterilized sawdust that was replaced daily. Cages were arranged on racks and their sanitation was continuously monitored. Biohazard bags [21] were used to autoclave infective biological materials at the end of the study (urine, feces, and carcass) [22].

### 2.6. Maintenance Diet and Supplied Water

Gerbils were given a commercial diet for a 20-day period based on their daily nutritional requirements. It was composed of casein, corn oil, fiber, mineral mixture, zinc gluconate, vitamins mixture, choline, corn starch, and sucrose [23]. Both diet and commercial pure water were supplied* ad libitum* to the gerbils throughout the entire study.

### 2.7. Culture, Preparation, and Administration of G. duodenalis Inoculum

The* Giardia *isolate used in this study was* G. duodenalis *strain GS/M83-H7.* Giardia *trophozoites culture (1 × 10^6^ trophozoites/mL, 250 *μ*L) was added in 15 mL glass screw-capped test tubes containing 7 mL of TYI-S-33 medium supplemented with 10% bovine serum. Subculturing was performed every 72–96 h by incubation in an anaerobic chamber (18% CO_2_ atmosphere) at 37°C for 24 h [24]. After incubation, the plates were placed on ice and rocked gently (20 min) to suspend nonadherent trophozoites, and the liquid was quickly discharged to avoid contamination using a purifier [25]. Trophozoites were washed 3 times in PBS pH 7.2 (GIBCO PBS pH 7.2, Life Technologies, Thermo Fisher Scientific Inc., Waltham, MA, USA) by centrifuging at 800 g for 10 min at 4°C. The pellet was resuspended in 500 *μ*L of PBS and further diluted 1 : 15 with PBS and 1 : 2 with 0.4% Trypan Blue (Solution Trypan Blue, SIGMA Aldrich Company, ST Louis MO, USA) for microscopic analysis and trophozoite counting in a Neubauer chamber at 40x magnification. Washed pellets were adjusted to a concentration of 5 × 10^6^ trophozoites in 200 *μ*L. Experimental infections with* Giardia *GS/M-83-H7 were established by orogastric inoculation with 5 × 10^6^ trophozoites suspended in 500 *μ*L of PBS [18].

### 2.8. Bioassay

Previous to the bioassay, the 42 female gerbils were weighed and gastric inoculation was performed with* G. duodenalis* trophozoites. A couple of inoculated gerbils were randomly selected, anaesthetized with chloroform, and euthanized for examination to confirm establishment of the infection via trophozoite detection in the duodenum and the proximal intestine at day 6 of postinfection [18]. Forty gerbils were randomly allocated into 5 treatment groups (8 per group). The treatments were randomly assigned to the 5 groups, and they comprised 0.5 mL oral doses of PBS (negative control), metronidazole (2 mg/mL in PBS or 1 mg per dose, positive control), or 1 of 3 different* Y. baccata* extracts with different concentrations (24.4 mg/mL, 12.2 mg/mL, and 6.1 mg/mL). Treatments were administered once daily for 3 days from day 7 of postinfection to day 9 of postinfection, and the weight of each gerbil was recorded again before euthanasia. After asepsis, 5 cm each of the proximal (midsection) and duodenum segments were removed and cut from fixed locations along the intestine length. Each segment was slit longitudinally and washed with PBS (shaking at 130 rpm) and the washing solutions were transferred to 3 mL vials and kept at 4°C until we counted the trophozoites. After homogenization, the solutions were transferred to conical tubes and centrifuged at 800 g at 4°C for 10 min. The supernatant was discharged and sediment was homogenized. An aliquot of the sediment (0.5 *μ*L) was diluted 1 : 20 with PBS, and 0.5 *μ*L of the dilution was placed in a Neubauer chamber. Trophozoites on each 1 mm² square (4 in total) were microscopically counted at 40x magnification (Microscope Olympus, CKX41, Center Valley, PA, USA). The total number of trophozoites per mL was calculated as follows: (Number of trophozoites/4 squares) × (500/100) × 10000 [26].

### 2.9. Study Design

**Figure 1:**
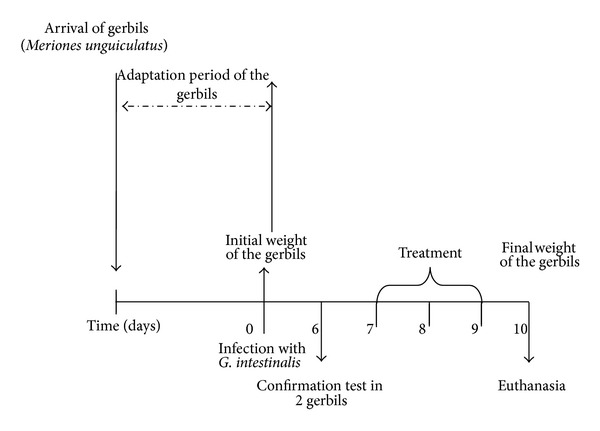
Design of the bioassay based on five treatments and 42 gerbils to test the antigiardial activity of the* Y. baccata* extracts.

This was a ten-day bioassay with a completely randomized design, in which the treatments consisted of different concentrations of* Yucca* extract and the experimental units were the gerbil groups infected with 5 × 10^6^ trophozoites of the* G. intestinalis* clone GS/M-83-H7. The assignment of gerbils to each experimental unit (groups) and the assignment of the treatments to each experimental unit (groups) were completely random (Figure 1).

### 2.10. Statistical Analysis

Descriptive statistics were generated for the weight and trophozoite count data from the study gerbils. The Wilcoxon matched-pairs signed rank test was used to compare the change of weight in the gerbil groups between the baseline and day 10 of postinfection. The effect of the 3 different concentrations of* Yucca's* extracts on* G. intestinalis* trophozoites were measured based on the geometric median of the active trophozoite counts observed in the intestinal tissue of the* Giardia* infected gerbils in each experimental unit after treatment, and the significance of the differences among log-transformed geometric medians was calculated with the nonparametric Kruskal-Wallis one-way and Tukey-Kramer (post hoc) tests. The Mann Whitney* U* test was used to test the difference in the geometric mean trophozoite counts between those from the duodenum and those from the proximal segments of the intestine of the infected gerbils. All analyses were performed using the STATA (Stata Corp LP, 4905 Lakeway Drive, College Station, TX 77845, USA), and statistical significance was established at *P* ≤ 0.05.

## 3. Results and Discussion

**Figure 2:**
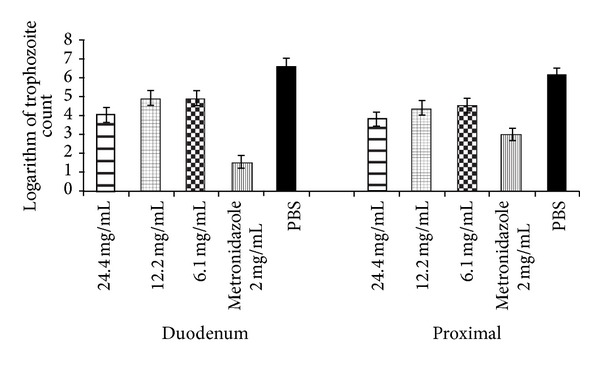
Effect of the extracts with 3 different concentrations of extracts per dose of* Y. baccata*, of metronidazole, and phosphate buffered saline (PBS) on the trophozoite counts in the duodenum and proximal of* Giardia* infected Mongolian gerbils.

This study investigated the activity of extracts of* Y. baccata* from the desert area of Northwestern Mexico against* G. duodenalis*. Our study gerbils were 6–12 weeks of age and weighed 52 to 56 grams. This weight and age combination is proper for bioassays in gerbils to test their susceptibility to* Giardia* infection. McAllister et al. [17] successfully developed bioassays using gerbils that were 6–9 weeks of age and weighing 50 to 60 g. In this study, infection with* G. duodenalis* in Mongolian gerbils (*Meriones unguiculatus*) was established by orogastric inoculation with 5 × 10^6^ trophozoites/500 *μ*L of PBS, and the test confirming infection in 2 gerbils at day 6 of postinfection revealed median trophozoite counts of 120,000 trophozoites/mL PBS and 85,000 trophozoites/mL PBS in the duodenum and the proximal segments, respectively. McAllister et al. [17] used 2 × 10^5^ *Giardia* trophozoites in 0.5 mL of PBS to infect, by orogastric inoculation, the same strain of gerbils as those used in this study. They found that, at day 6 after inoculation, the trophozoite counts tended to be higher in the duodenum than those in the jejunum or ileum. In this study, the trophozoite counts always tended to be higher in the duodenum than those in the proximal intestine of the infected gerbils (*P* ≥ 0.05) (data not shown). Most likely, the different configuration of the intestinal membrane at the bottom of the villus and the villus : crypt ratios play an important role in trophozoite attachment in the different sections of the small intestine sections [27]. The median weights at preinfection (baseline) and at day 10 of postinfection for 31 gerbils randomly allocated to 5 experimental groups are shown in Table 1. Over the course of the study, each experimental group showed weight loss, but it was not significant (Table 1). Bénéré et al. [28] published that the overall weight gain in 24 gerbils was significantly lower for 16* Giardia* infected gerbils (0.491 ± 0.0167 g) than 8 gerbils free of* Giardia intestinalis* (0.769 ± 0.059 g) at day 18 of postinfection. It is well recognized that giardiosis may be asymptomatic or associated with acute and chronic diarrhea.* Giardia* trophozoites may cause intestinal lesions, leading to nutrient malabsorption that may explain the weight loss in infected humans [29] or animals [30]. Most likely, this 10-day bioassay was not sufficiently long to observe differences in the pre- and postinfection gerbil weights. In addition, the orogastric administration of the 24.4 mg/mL, 12.2 mg/mL, and 6.1 mg/mL butanol extracts did not significantly reduce the trophozoite counts in the duodenum segment of the infected gerbils compared to the untreated group at day 10 of postinfection. In addition, no difference was observed in the trophozoite count reductions among the 3 extract-treated groups (*P* ≥ 0.05) (Figure 2).

In contrast, the high concentration extract (24.4 mg/mL) alone significantly reduced (*P* < 0.05) the trophozoite counts in the proximal segment of the infected gerbils compared to the untreated infected gerbils (Figure 2). There was no difference in the trophozoite count reductions between the group treated with the high extract concentration and the group treated with metronidazole (*P* > 0.05). The trophozoite count was significantly lower for the high concentration extract (*P* < 0.05) than that for the medium and low extract concentrations (Figure 2). The geometric mean of the trophozoite counts remained unchanged in the untreated infected gerbils at day 10 of postinfection in both the duodenum and proximal segments (Figure 2). In contrast, metronidazole significantly reduced the trophozoite counts in both the upper and lower intestine of the infected gerbils (*P* = 001) (Figure 1). McAllister et al. [17] found that the oral administration of 6.1 mg/mL of* schidigera* extracts using the same treatment regime as that used in the present study significantly reduced the number of trophozoites in the lower region (jejunum and ileum) but not the number in the duodenum of the infected gerbils. Those authors explained that the extract's compounds may form complexes with bile salts released in the duodenum [17, 31, 32], reducing their antigiardial properties in the upper regions of the small intestine. However, only the higher extract concentration was capable of significantly reducing the trophozoite counts in the lower region. Gerbils exhibited toxic reactions to the different administered concentrations in this study. Three gerbils per group that received* Yucca* extracts died. McAllister et al. [17] found that a higher extract concentration than the concentrations in this study (50 mg of butanol extract in eight doses over the 3 day period) had a negative impact on the overall health of the gerbils. Although metronidazole failed to eradicate the trophozoite populations, it was more effective at eliminating trophozoites from the proximal segment than from the duodenum segment of the intestine.

## 4. Conclusion

The butanol extracts of* Y. baccata* exhibited antigiardial activity by reducing, although not significantly, the trophozoite counts in the duodenum, and the higher extract concentration showed a similar antigiardial activity to that of metronidazole in the proximal segment. The effective antigiardial activity in the proximal segment and the toxic effects observed in the gerbils in this study have encouraged us to study the chemical characterization, mechanism of action, and degree of toxicity of the components with antigiardial properties and to compare their antigiardial efficacy against* Y. schidigera *under similar laboratory conditions. Extracts of* Y. baccata* may represent an effective and economical alternative to treat intestinal parasitism in humans.

## Figures and Tables

**Table 1:** Median weight of 35 gerbils allocated in 5 treatment groups at preinfection and day 10 of postinfection during the bioassay.

Butanol extract	*n*	Weight at preinfection (g) Median (SE)	Weight at day 10 of postinfection (g) Median (SE)	*n*	ΔWeight (g)	P*
24.4 mg/mL of extract^&^	5	52.7 (1.44)	50.3 (1.84)	5	−2.4	0.293
12.2 mg/mL of extract^&^	5	48.7 (3.1)	47.5 (3.87)	5	−1.2	0.403
6.1 mg/mL of extract^&^	5	56.6 (1.2)	55.6 (1.18)	5	−1.0	0.400
Metronidazole	8	56.0 (2.71)	55.3 (2.62)	8	−0.7	0.636
Untreated	8	52.6 (2.25)	49.9 (2.21)	8	−2.7	0.372

(SE): (Standard error); *P*: ∗Wilcoxon matched-paired-sum test; Significance at *P* ≤ 0.05.

^&^3 gerbils died per extract-treated group during the bioassay course.
